# In Vitro Cell Proliferation, Osteogenic Differentiation, and In Vivo Angiogenesis Induced by Modified mRNA Encoding Vascular Endothelial Growth Factor‐A

**DOI:** 10.1155/ijod/8805818

**Published:** 2026-10-14

**Authors:** Noppadol Sa-Ard-Iam, Mana Naratippakorn, Chantrakorn Champaiboon

**Affiliations:** ^1^ Immunology Research Center Faculty of Dentistry Chulalongkorn University Bangkok Thailand chula.ac.th; ^2^ Center of Excellence in Periodontal Disease and Dental Implant Chulalongkorn University Bangkok Thailand chula.ac.th; ^3^ Department of Periodontology Faculty of Dentistry Chulalongkorn University Bangkok Thailand chula.ac.th

**Keywords:** angiogenesis, CAM assay, mRNA, periodontal regeneration, SDG3, vascular endothelial growth factor, VEGF

## Abstract

**Aims:**

This study aimed to determine whether pseudouridine‐modified mRNA encoding vascular endothelial growth factor (VEGF) ‐A induces VEGF production in human periodontal ligament cells (PDLCs), and to evaluate its effects on PDLC proliferation, osteogenic differentiation, and in vivo angiogenesis.

**Methods:**

PDLCs derived from healthy periodontal tissues were transfected with pseudouridine‐modified VEGF‐A mRNA using Lipofectamine 2000 (L2000) or treated with L2000 alone. VEGF secretion was quantified at 24–72 h posttransfection. Cell viability and immune activation were also assessed. Supernatants collected at 48 h were used to evaluate PDLC proliferation using the AlamarBlue assay. Osteogenic differentiation was assessed by Alizarin Red S staining and the expression of *alkaline phosphatase gene* (*ALPL*), *RUNX2*, and *osteocalcin* (*OCN*). Angiogenic activity was evaluated using the chorioallantoic membrane (CAM) assay in fertilized White Leghorn chicken eggs (*n* = 7 per group). On embryonic day 8, filter papers loaded with supernatants from L2000 + mRNA, L2000 groups, Dulbecco’s phosphate‐buffered saline, or recombinant VEGF (rhVEGF) were placed on the CAM. After a 3‐day incubation, newly formed blood vessels were imaged and quantified.

**Results:**

PDLCs transfected with pseudouridine‐modified VEGF‐A mRNA produced significantly higher VEGF levels than the control groups for up to 72 h (*p* < 0.05), without affecting cell viability or inducing innate immune activation. VEGF‐containing supernatants promoted PDLC proliferation. Direct VEGF mRNA transfection enhanced osteogenic differentiation and upregulated expression of osteogenic markers *ALPL*, *RUNX2*, and *OCN*. In the CAM assay, abundant microvascular networks with a characteristic spoked‐wheel pattern were observed in both the VEGF mRNA and rhVEGF groups. Furthermore, the VEGF mRNA group showed a significantly greater increase in blood vessel formation than the control groups (*p* < 0.01).

**Conclusion:**

Pseudouridine‐modified VEGF‐A mRNA effectively induced VEGF production, which subsequently enhanced PDLC proliferation, osteogenesis, and in vivo angiogenesis. These findings support the potential of pseudouridine‐modified VEGF‐A mRNA as a promising therapeutic approach for periodontal regeneration.

## 1. Introduction

The mRNA platform has emerged as a promising therapeutic strategy for disease prevention and treatment, with applications in genetic, cardiovascular, and neurodegenerative disorders, as well as the development of COVID‐19 vaccines. Compared with DNA‐based therapies, the mRNA platform provides significant advantages, including the absence of genomic integration and direct cytoplasmic protein expression without requiring nuclear entry [1, 2]. However, its therapeutic application has historically been limited due to rapid degradation by RNases, low translational efficiency, and activation of innate immune responses through recognition by pattern‐recognition receptors [3–5].

Recent advances in mRNA engineering have largely overcome these limitations. Incorporation of chemically modified nucleosides, particularly replacing uridine with either pseudouridine or N‐1 methylpseudouridine, has exhibited markedly attenuated immunogenicity while simultaneously enhanced protein translation efficiency [6–8]. Various delivery approaches have been developed to protect mRNA from degradation and enhance the efficiency of protein production. These emerging gene delivery systems include polymer‐, peptide‐, and lipid‐based carriers [9–12]. Among these systems, cationic lipid nanoparticles are one of the most reliable platforms due to their ease of synthesis and efficient cellular uptake [13, 14]. Furthermore, mRNA stability and translation efficiency can be enhanced by optimization of structural elements such as the 5^′^ cap structure, untranslated regions (UTRs), poly(A) tail length, and modified coding sequence [15].

Vascular endothelial growth factors (VEGFs) comprise a family of homodimeric proteins including VEGF‐A, VEGF‐B, VEGF‐C, VEGF‐D, and placental growth factor [16]. Among these, VEGF‐A is the most abundant and well‐characterized isoform and serves as a key regulator of angiogenesis. It promotes endothelial cell proliferation, migration, and activation as well as providing prosurvival effects on vascular, endothelial, and cardiac cells [16]. VEGF‐A has been delivered as recombinant protein, naked plasmid DNA (pDNA), and adenoviral vectors to accelerate impaired wound healing [16–21]. Clinical studies have demonstrated that VEGF has been used for treating chronic neuropathic lower‐extremity diabetic ulcers, pressure ulcers, and postoperative wounds [17, 18, 21]. In addition to direct angiogenic activity, VEGF also promotes bone regeneration [22] and reciprocates adipogenic differentiation [23].

VEGF‐A is considered a promising growth factor for facilitating periodontal regeneration due to its potent angiogenic properties [24]. However, the clinical efficacy of recombinant growth factors for periodontal regeneration remains inconclusive [25]. The major downfall of using recombinant proteins is their short in vivo half‐life, which typically ranges from several hours to a few days [26]. Consequently, supraphysiologic doses or repeated administrations are often required to maintain efficacy, which increases costs and the risk of adverse effects [27].

The therapeutic potential of chemically modified VEGF‐A mRNA has been demonstrated in several preclinical models of tissue regeneration. In a murine model of ischemic heart disease, administration of 5‐methylcytidine and pseudouridine‐modified VEGF‐A mRNA enhanced vascularization, reduced infarct size, and improved survival rates compared to pDNA‐transfected and control groups [28]. Similarly, intracardiac injection of an optimized VEGF‐A mRNA improved cardiac function in a swine model of myocardial infarction [29]. In a murine diabetic model, intradermal delivery of VEGF‐A mRNA accelerated tissue healing by shortening the time to achieve 50% wound closure by ~5 days [30].

More recently, the clinical application of VEGF‐A mRNA therapy has been explored. In a Phase I study involving patients with type 2 diabetes mellitus, the intradermal administration of modified VEGF‐A mRNA induced local VEGF‐A expression and a transient increase in skin blood flow [31]. Furthermore, a randomized clinical trial evaluating the direct intramyocardial injection of naked VEGF mRNA in seven patients undergoing coronary artery bypass surgery reported no serious adverse events or clinical immune reactions and a promising trend toward improved left ventricular ejection fraction [32, 33]. Collectively, these preclinical and early clinical investigations support the feasibility of VEGF mRNA therapy for promoting angiogenesis and tissue regeneration. However, its therapeutic efficacy across various regenerative applications, including periodontal regeneration, remains limited.

The chorioallantoic membrane (CAM) assay is a well‐established in vivo model for evaluating angiogenic activity due to its simplicity, rapid development, and cost‐effectiveness. The CAM is an extraembryonic membrane formed by the fusion of the chorion and allantois, which serves as a highly vascularized gas‐exchange surface. CAM development begins around embryonic day 3 (E3) and expands rapidly until around embryonic day 10 (E10) [34]. Since the embryonic immune system is not fully developed until approximately embryonic day 18, this model enables the evaluation of a wide range of test substances with minimal risk of immune rejection [35].

Given the therapeutic potential of mRNA‐based strategies, this study aimed to develop highly translating, pseudouridine‐modified mRNA encoding VEGF‐A and evaluate its translation and secretion efficiency in human periodontal ligament cells (PDLCs), the primary target cells for periodontal regeneration. Although modified VEGF‐A mRNA has been investigated in other regenerative applications, its use in human PDLCs (hPDLCs) for periodontal regeneration has not previously been explored. The second objective was to determine whether the secreted VEGF‐A could promote cell proliferation and osteogenic differentiation in vitro. Furthermore, the CAM assay was employed to assess the angiogenic activity of the translated VEGF‐A in vivo. The findings provide proof of concept for a modified VEGF‐A mRNA platform in periodontal regenerative research and may facilitate future in vivo investigations.

## 2. Methods

### 2.1. The Construction of Chemically Modified mRNA Encoding VEGF‐A

The nucleotide sequence of pseudouridine (*Ψ*)‐modified mRNA encoding VEGF‐A was designed as shown in Figure 1 and submitted to TriLink BioTechnologies (San Diego, CA, USA). The construction of modified mRNA encoding VEGF‐A with cap1, followed by DNase and phosphatase treatment and silica‐membrane purification, was supplied as a solution in 1 mM sodium citrate (pH 6) [36].

**Figure 1:**
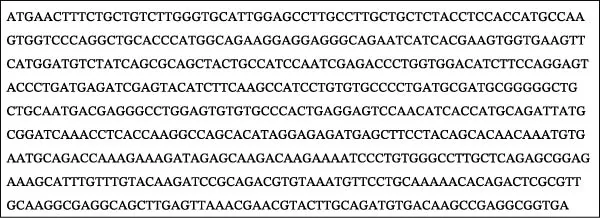
Pseudouridine (*Ψ*)‐modified VEGF‐A gene sequence [36].

### 2.2. Cell Isolation

hPDLCs were isolated from the periodontal ligament tissue obtained from patients treated at the Graduate Periodontics Clinic or the Department of Oral and Maxillofacial Surgery, Faculty of Dentistry, Chulalongkorn University. Heparinized peripheral blood samples were collected from healthy adult volunteers through the Thai Red Cross Society.

#### 2.2.1. hPDLCs

PDLCs were harvested from extracted teeth due to therapeutic reasons. PDLCs were isolated using the enzyme digestion method [36, 37]. Briefly, the extracted tooth was thoroughly washed twice with Dulbecco’s phosphate‐buffered saline (DPBS; Gibco, Thermo Fisher Scientific, Grand Island, NY, USA), and PDL tissue was scraped out from the mid‐root to avoid contamination from gingival or periapical tissues. Furthermore, the tissue was minced into 1–2 mm^2^ fragments and digested with a solution containing 2 mg/mL collagenase (Sigma–Aldrich, St. Louis, MO, USA) for 60 min at 37°C. The cell suspension was filtered through a 70‐µm cell strainer (Falcon; Corning Incorporated, Corning, NY, USA). The pass‐through was washed twice with minimum essential medium with alpha modification (*α*‐MEM; Gibco). PDLCs were cultured with *α*‐MEM supplemented with 10% heat‐inactivated fetal bovine serum (Gibco) and 1% antibiotic‐antimycotic solution (Gibco) at 37°C in a humidified atmosphere of 5% CO_2_. The medium was replaced twice a week. Cells from the 3^rd^ to 8^th^ passages obtained from four different donors were used in this study [36]. All in vitro experiments were performed using independent biological replicates derived from these four donors.

#### 2.2.2. Human Peripheral Blood Mononuclear Cells (PBMCs)

PBMCs were isolated as previously described by Böyum [38]. Briefly, heparinized blood was layered over Ficoll‐hypaque (Sigma–Aldrich) and centrifuged at 700 × g for 30 min at 25°C. PBMCs were washed twice and resuspended in RPMI 1640 medium (Gibco) supplemented with 10% heat‐inactivated fetal bovine serum.

### 2.3. Assessment of In Vitro VEGF‐A Production

Pseudouridine (*Ψ*)‐modified mRNA encoding VEGF‐A (VEGF mRNA) was complexed with Lipofectamine 2000 (L2000; Invitrogen, Life Technologies, Carlsbad, CA, USA) according to the manufacturer’s instructions. PDLCs (1 × 10^5^ cells/well) were transfected with L2000 + VEGF mRNA complexes (1 μg of modified VEGF mRNA combined with 1.5 μL of L2000) or with 1.5 μL of L2000 alone in a final volume of 50 μL. The transfection was carried out for 4 h at 37°C, and then the medium was replaced with *α*‐MEM medium. The transfected cells were cultured for another 24–72 h, and then both cell lysates and supernatants were collected and subjected to analysis of VEGF production using an enzyme‐linked immunosorbent assay (ELISA) kit (Quantikine, R&D Systems, Minneapolis, MN, USA).

### 2.4. Assessment of Cell Viability and Immune Activation After Transfection

Cell viability was assessed using the AlamarBlue assay. Briefly, transfected cells were incubated with 10% AlamarBlue solution (Bio‐Rad Laboratories, Hercules, CA, USA) at 37°C under humidified conditions with 5% CO_2_. The supernatants were collected at 24, 48, and 72 h posttransfection. Optical density was measured at 570 and 600 nm using a microplate reader (Epoch Microplate Spectrophotometer, BioTek Instruments Inc., Winooski, VT, USA). To determine whether VEGF mRNA induces innate immune activation, PBMCs derived from four independent individuals were transfected with L2000 alone or L2000 + VEGF mRNA for 24–48 h. The levels of proinflammatory cytokines, for example., interferon (IFN)*-α* and tumor necrosis factor (TNF)‐*α*, were subsequently quantified using (ELISA) kits (Quantikine).

### 2.5. Stimulation of Human PDLC Proliferation

To evaluate the effect of VEGF mRNA on PDLC proliferation, fresh hPDLCs were seeded in 96‐well plates and cultured overnight. Either the supernatants of L2000 + VEGF mRNA, L2000 alone, or control medium (2.5 ng/100 μL/well) were added to the culture. After 48 h of incubation, 10% AlamarBlue solution was added and incubated for an additional 4 h. The culture supernatants were collected, and optical density was measured at an absorbance of 570 and 600 nm using a microplate reader.

### 2.6. Assessment of In Vitro Osteogenic Differentiation

#### 2.6.1. Alizarin Red S Staining

To evaluate the effect of the VEGF mRNA transfection on osteogenesis in vitro. Alizarin Red S staining was performed as described by Geng et al. [39]. In brief, hPDLCs were cultured in 24‐well plates and transfected with L2000 + 1 μg of modified VEGF mRNA, L2000 alone, or control medium. After 4 h, the culture medium was switched to an osteoinductive medium consisting of complete medium supplemented with 50 µg/mL L‐ascorbic acid phosphate magnesium salt, 10 mM *β*‐glycerophosphate, and 10 nM dexamethasone (Sigma–Aldrich); the medium was changed every 3–4 days. After 21 days, the cells were rinsed with phosphate‐buffered saline and fixed with 10% formalin at room temperature for 10 min. The cells were then washed with distilled water and treated with a 1% Alizarin Red S solution (pH 4.1–4.3; Sigma–Aldrich) for 3 min. The cells were thoroughly washed to remove excess staining. Mineralized nodules were subsequently analyzed and imaged under a light microscope (Olympus Life Science, Tokyo, Japan). For quantitative analysis of mineral deposition, Alizarin Red S‐stained cells were incubated with 10% cetylpyridinium chloride (Sigma–Aldrich) in 10 mM sodium phosphate buffer (pH 7.0) for 15 min to solubilize the bound stain. The solution was transferred to 96‐well plates, and the absorbance was measured at 562 nm using a microplate reader.

#### 2.6.2. Alkaline Phosphatase (ALP) Activity

Seven days post‐osteogenic induction, transfected and nontransfected cells were washed with PBS, and ALP activity was measured using a Lab Assay ALP (Fujifilm Wako Pure Chemical Corp., Chuo‐Ku, Osaka, Japan) according to the manufacturer’s instructions. The absorbance was measured at 405 nm with a microplate reader.

#### 2.6.3. qRT‐PCR

Seven days post‐osteogenic induction, total RNA was isolated from transfected and nontransfected hPDLCs using an RNeasy Mini Kit (Qiagen, Hilden, Germany). One microgram of DNase I‐treated total RNA was reverse‐transcribed using the SuperSctipt VILO cDNA synthesis Kit (Invitrogen, California, USA). The cDNA was used for PCR amplification of osteogenic‐related gene expression levels. qRT‐PCR assays were performed using a LightCycler 480 System (Roche Molecular Diagnostics, Mannheim, Germany) with the SYBR Green PCR Master Mix (Roche Molecular Diagnostics). The primer sequences for human *alkaline phosphatase gene* (*ALPL*), *RUNX2*, *osteocalcin* (*OCN*), *and GAPDH* genes are listed in Surisaeng et al. [40]. The PCR cycling parameters were 95°C for 10 min and 45 cycles of 95°C for 10 s, 60°C for 10 s, and 72°C for 20 s. Gene expression levels were normalized to *GAPDH*, which served as an internal control according to the 2^−ΔΔCt^ method.

### 2.7. The CAM Assay

To evaluate the angiogenic potential of VEGF mRNA transfection, the CAM assay was performed. All animal procedures were conducted at the Immunology Research Center, Faculty of Dentistry, under the approval of the Institutional Animal Care and Use Committee (IACUC) of the Faculty of Veterinary Science, Chulalongkorn University, Thailand (Approval Number 2031021). A sample size calculation was performed using 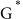
Power (*F* tests, one‐way ANOVA: fixed effects, omnibus). Based on the expected group means reported by Rumney et al. [41], an effect size of Cohen’s *f* = 0.72 was estimated. With *α* = 0.05 and 80% power, the minimum required sample size was 28 embryos (7 per group).

Fertilized White Leghorn chicken eggs were obtained from Kasetsart University, Bangkok, Thailand. The eggshell was cleaned with 70% ethanol and incubated at 37°C with 60% humidity. On embryonic day 3 (E3), a windowing procedure was performed. Briefly, ~3 mL of albumen was aspirated using a syringe fitted with a 25‐gauge blunt‐end needle to detach the shell membrane. A 10 mm × 20 mm window was then created in the eggshell using small dissecting scissors, and sealed with transparent tape, and the egg was returned to the incubator.

On day 8 of incubation (E8), the eggs were randomly allocated into four treatment groups. Filter paper discs (5 mm in diameter; Whatman filter, Grade 41, 220 μm thick, pore size 20–25 μm; Sigma–Aldrich, Dorset, UK) were soaked with DPBS, recombinant human VEGF (500 ng/disc) (recombinant VEGF [rhVEGF]; R&D Systems, Abingdon, UK), L2000, or L2000 + VEGF mRNA supernatants and individually placed onto the CAM surface through the window. The window was resealed with transparent tape, and the egg was incubated for an additional 3 days [36, 41–43].

The CAM was photographed and counted under a stereomicroscope (Olympus SZ61, Tokyo, Japan) at E8 and E11 [36, 42, 43]. All images were acquired with the filter disc positioned at the center of the field of view to ensure a standardized image acquisition. Images were processed using the automated Otsu’s gray‐thresholding method in ImageJ software (National Institutes of Health, Bethesda, MD, USA). The region of interest (ROI) was defined as a 1‐mm zone surrounding the disc. The total number of microvessels (3–10 μm in diameter) converging toward the disc was counted at x 10 magnification. An increase in the number of microvessels from E8 to E11 indicated an angiogenic response [36, 42, 43]. Vessel counting was performed independently in a blinded manner by three observers who were not aware of the treatment allocation. Interexaminer reliability was excellent, with an intraclass correlation coefficient (ICC) greater than 0.90.

### 2.8. Statistical Analysis

Statistical analyses were performed using data obtained from four independent biological donors and analyzed with IBM SPSS Statistics 29.0 (IBM Corp., Armonk, NY, USA). Data normality was assessed using the Shapiro–Wilk test. As the data were not normally distributed, differences among groups were analyzed using the nonparametric Kruskal–Wallis test. When significant differences were identified, pairwise comparisons were performed using the Bonferroni‐adjusted Mann–Whitney *U* test. VEGF production at each time point and CAM assay data were analyzed using the Kruskal–Wallis test, followed by Bonferroni‐adjusted Mann–Whitney *U* tests for pairwise comparisons. A *p*‐value less than 0.05 was considered statistically significant.

## 3. Results

### 3.1. In Vitro VEGF‐A Production

To evaluate and optimize pseudouridine‐modified mRNA delivery into hPDLCs using L2000, transfection efficiency was first assessed using pseudouridine‐modified green fluorescent protein (GFP) mRNA as a reporter. Flow cytometry and fluorescence microscopy at 24 h demonstrated efficient intracellular GFP expression, confirming successful mRNA delivery and a high transfection efficiency of ~93% in hPDLCs (Figure 2A).

**Figure 2:**
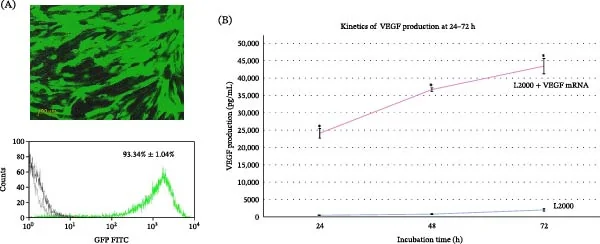
VEGF‐A production by human PDLCs following transfection with modified VEGF‐A mRNA. (A) Assessment of transfection efficiency using pseudouridine‐modified GFP mRNA by fluorescence microscopy and flow cytometry at 24 h. (B) Human PDLCs were transfected with modified VEGF‐A mRNA complexed with Lipofectamine 2000 (L2000 + VEGF mRNA) or Lipofectamine 2000 alone (L2000). VEGF‐A levels in the supernatants collected at 24, 48, and 72 h posttransfection were quantified by ELISA. Data are presented as mean ± SE (*n* = 4 independent donors).  ^∗^, *p* < 0.05.

In vitro transfection with pseudouridine‐modified VEGF‐A mRNA induced VEGF production in hPDLCs. The levels of VEGF protein were detected in both cell lysates and supernatants. The mean concentration of VEGF in the supernatants was 25,105 ± 1326.84 in the L2000+VEGF mRNA group, compared with 545.93 ± 25.69 in the L2000 group at 24 h. VEGF levels in the L2000+VEGF mRNA group were significantly higher than those in the L2000 control (*p* = 0.029; Figure 2B). Notably, VEGF production in the sham‐transfected (L2000) group was comparable to that in the negative control group. Furthermore, extracellular VEGF protein levels in the VEGF mRNA group increased over time from 24 to 72 h and were consistently higher than those in the L2000 group at all time points (*p* = 0.029; Figure 2B).

### 3.2. Cell Viability and Immune Activation Following Transfection

Cell viability following modified VEGF‐A mRNA transfection in hPDLCs was assessed using the AlamarBlue assay. At 24 h posttransfection, cell viability was 88.24% ± 4.17% in the L2000 + VEGF mRNA group and 97.89% ± 0.74% in the L2000 group, relative to nontransfected controls (100%). No significant differences were observed among the groups. Moreover, cell viability in the L2000 + VEGF mRNA group remained above 90% for up to 72 h posttransfection (Figure 3A).

**Figure 3:**
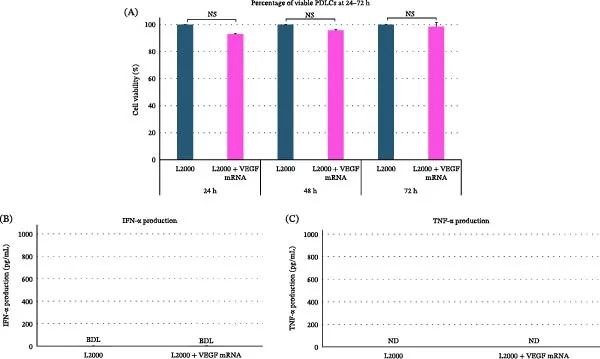
Effects of modified VEGF‐A mRNA transfection on cell viability and innate immune activation. (A) Viability of human PDLCs at 24, 48, and 72 h following transfection with modified VEGF‐A mRNA. Data are presented as mean ± SE (*n* = 4 independent donors). (B) Interferon (IFN)‐*α* and (C) tumor necrosis factor (TNF)‐*α* production by peripheral blood mononuclear cells (PBMCs) following VEGF‐A mRNA transfection at 24 and 48 h, as determined by ELISA (*n* = 4 independent donors). BDL, below detection limit; ND, not detected; NS, not significant.

Innate immune activation following VEGF mRNA transfection was further evaluated using PBMCs. Supernatants from transfected cells induced only negligible IFN‐*α* levels (Figure 3B) and did not stimulate TNF‐*α* production (Figure 3C).

### 3.3. Stimulation of Human PDLC Proliferation

Next, the proliferative effect of supernatants derived from modified VEGF‐A mRNA‐transfected cultures was assessed in hPDLC. Treatment with these supernatants significantly increased PDLC proliferation by up to 1.6‐fold compared with the control and L2000 groups (*p* < 0.05; Figure 4A).

**Figure 4:**
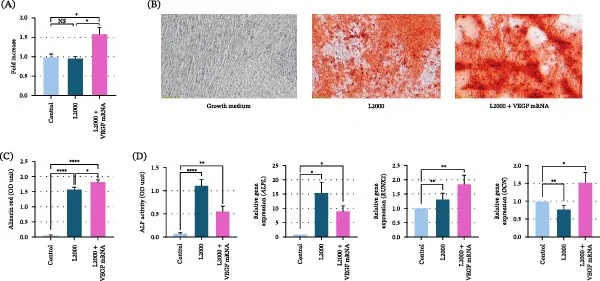
Effects of modified VEGF‐A mRNA transfection on PDLC proliferation and osteogenic differentiation. (A) Human PDLCs were treated with supernatants from cells transfected with Lipofectamine 2000 alone (L2000) or Lipofectamine 2000‐VEGF‐A mRNA complex (L2000 + VEGF mRNA) and compared with untreated controls. Cell proliferation was evaluated after 48 h of incubation (*n* = 4 independent donors). (B) Osteogenic differentiation of hPDLCs following direct transfection with modified VEGF‐A mRNA. Alizarin Red S staining was performed 21 days posttransfection in hPDLCs treated with growth medium, L2000, or L2000 + VEGF mRNA. Images are representative of four independent experiments. (C) Quantitative analysis of mineral deposition based on Alizarin Red S staining following modified VEGF‐A mRNA transfection. (D) Alkaline phosphatase (ALP) activity and relative expression levels of osteogenic marker genes *ALPL*, *RUNX2*, and *osteocalcin* (*OCN*) following modified VEGF‐A mRNA transfection (*n* = 4 independent donors).  ^∗^, *p* < 0.05;  ^∗∗^, *p* < 0.01;  ^∗∗∗∗^, *p* < 0.0001; NS, not significant.

### 3.4. In Vitro Osteogenic Differentiation of Human PDLCs

The effect of in vitro transfection with modified VEGF‐A mRNA on osteogenic differentiation was evaluated using Alizarin Red S staining. VEGF mRNA transfection induced osteogenic differentiation in hPDLCs and markedly promoted focal mineralization at day 21 compared with the control group (growth medium) (Figure 4B). Quantitative analysis of Alizarin Red S staining confirmed a statistically significant increase in mineral deposition in the VEGF mRNA‐transfected group compared with the L2000 group (*p* < 0.05; Figure 4C).

Furthermore, transfection with modified VEGF‐A mRNA significantly upregulated ALP enzymatic activity in hPDLCs compared with control groups (*p* < 0.01; Figure 4D). In alignment with the enhancement of cellular mineralization and enzymatic activity, real‐time quantitative PCR analysis revealed that modified VEGF mRNA transfection significantly upregulated the mRNA expression levels of key osteogenic marker genes, including *ALPL*, *RUNX2*, and *OCN*, relative to the control (*p* < 0.05; Figure 4D).

### 3.5. Induction of Angiogenesis in the CAM Assay

The biological activity of the translated VEGF‐A protein present in the supernatants was assessed for its ability to induce blood vessel formation in vivo using the CAM assay. At E8, only a few microvessels were observed in the CAM across all treatment groups (Figure 5A; two left panels). By E11, more abundant microvascular networks with a characteristic spoked‐wheel pattern were observed in both L2000+VEGF mRNA and rhVEGF groups, whereas this pattern did not appear in the control groups (Figure 5A; two right panels). At E8, the mean numbers of microvessels were 22.62 ± 1.42 in the L2000+VEGF mRNA group, 23.38 ± 1.65 in the rhVEGF group, 24.48 ± 2.26 in the L2000 group, and 20.81 ± 2.14 in the DPBS control group. From E8 to E11, the mean increases in the microvessel number were 51.38 ± 2.23 in the L2000+VEGF mRNA group and 42.86 ± 5.62 in the rhVEGF group, compared with 32.71 ± 1.97 in the L2000 group and 31.30 ± 3.00 in the DPBS group. The L2000+VEGF mRNA group exhibited a significantly greater increase in microvessel formation than both the DPBS and L2000 groups (*p* = 0.003 and 0.006, respectively; Figure 5B).

**Figure 5:**
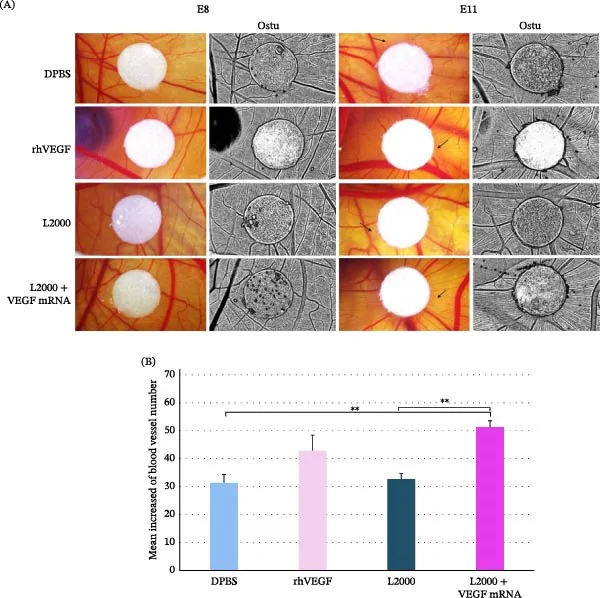
In vivo angiogenesis assessed by the CAM assay. (A) Representative stereomicroscope images and corresponding images generated using Otsu’s thresholding method at embryonic day 8 (E8) and day 11 (E11) following placement of Whatman filter discs soaked with DPBS, recombinant human VEGF (rhVEGF), supernatants from the L2000 group, or supernatants from the L2000 + VEGF mRNA group. Spoked‐wheel patterns of microvascular networks are indicated by black arrows (original magnification, 10 × ). (B) Mean increase in blood vessel number from E8 to E11 in the DPBS, rhVEGF, L2000, and L2000 + VEGF mRNA groups. Data are presented as mean ± SE (*n* = 7).  ^∗∗^, *p* < 0.01.

## 4. Discussion

This study explored the potential application of a modified pseudouridine nucleotide VEGF mRNA platform for periodontal regeneration. We investigated the in vitro transfection efficiency, cytotoxicity, proliferative, angiogenic, and osteogenic effects of chemically modified VEGF‐A mRNA complexed with a cationic lipid in hPDLCs, which are key cells involved in periodontal regeneration. The incorporation of the pseudouridine nucleotide along with optimized coding sequences and stringent purification by high‐performance liquid chromatography enhances mRNA stability, reduces immunogenicity, and enables transient protein expression [7]. Our findings demonstrated that transfection of modified pseudouridine VEGF‐A mRNA resulted in sustained VEGF secretion for up to 72 h, with scarce effects on cell viability and negligible innate immune activation. Furthermore, the translated VEGF promoted hPDLC proliferation and osteogenic activity while enhancing microvessel formation in the CAM assay.

The advantages of the mRNA transfection platform include the absence of genomic integration risk, no requirement for immunogenic vectors, high translational efficiency independent of the cell cycle, and controllable protein expression without gene overexpression [44]. In addition, mRNA is directly translated within the cytoplasm and can be expressed within minutes after transfection, thereby eliminating the need for nuclear entry and transcription [45].

Cell viability and enhanced cell proliferation are key factors in the safety and efficacy of biomaterials for clinical use. Furthermore, the possibility of triggering an inflammatory response needs to be taken into consideration. Earlier generations of mRNA therapies were reported to elicit an innate immune response, resulting in undesirable inflammation and aberrant protein production; however, this drawback had been overcome through chemical modification of mRNA [8]. Substituting uridine with pseudouridine, in particular, suppresses mRNA‐mediated immune activation while simultaneously enhancing protein translation efficiency [7, 8]. Consistent with these findings, our results revealed that the pseudouridine‐modified‐VEGF‐A mRNA complexed with L2000 had negligible effects on cell viability and innate immune activation. Furthermore, supernatants collected from transfected cells promoted hPDLC proliferation.

An adequate blood supply is critical for successful periodontal regeneration. Angiogenesis involves endothelial cell migration and proliferation, capillary tube formation, and subsequent mesenchymal stem cell stabilization of newly formed vessels [46]. VEGF is commonly considered one of the most potent mediators regulating vascular endothelial function [47]. VEGF‐A has been extensively investigated as a therapeutic agent to improve myocardial outcomes following ischemic injury. Furthermore, modified mRNA mediates “pulse‐like” expression of VEGF‐A and exhibited superior efficacy compared with pDNA, significantly reducing infarct size, enhancing myocardial perfusion, and improving survival in a murine model of myocardial infarction [28].

Additionally, modified VEGF mRNA‐engineered human adipose‐derived stem cells (hADSCs) cotransplanted with human fat have been used for soft tissue reconstruction and rejuvenation in a murine model. This modified VEGF enhanced graft survival and promoted cell differentiation [48]. Furthermore, VEGF translated from mRNA transfection stayed longer than rhVEGF‐A application. The half‐life of intravenously administered rhVEGF is relatively short (~30 min) [49], whereas our study revealed that hPDLCs stimulated with VEGF‐A mRNA produced and secreted high levels of VEGF for up to 72 h. Consistently, an in vitro study in which modified VEGF‐A mRNA was transfected into human cardiac endothelial intermediates showed peak VEGF production within 24 h and sustained for at least 3 days [50]. In our study, supernatants from the transfected cells stimulated hPDLC proliferation, and the transfected PDLCs subsequently promoted osteogenic differentiation.

PDLCs were selected because of their stemness potential and capacity to differentiate into various cell types, including osteoblasts and cementoblasts, which are crucial for periodontal regeneration [51]. In our laboratory, the mRNA transfection platform has been applied to a panel of growth factor mRNAs, including platelet‐derived growth factor‐BB (PDGF‐BB) and bone morphogenetic protein‐2 (BMP‐2) [40]. Our results revealed that PDLCs or gingival fibroblast cells transfected with each modified growth factor mRNA exhibited robust protein expression within 24 h. The mRNA platform permits rapid sequence modification of various genes and provides a simple and effective approach for generating different growth factors to support periodontal regeneration.

For proliferation and angiogenesis assays, conditioned medium collected from VEGF mRNA‐transfected PDLCs was used to evaluate the paracrine effects of secreted VEGF, which may influence surrounding cells and tissues in a therapeutic setting. In contrast, for the osteogenic differentiation assay, PDLCs were directly transfected with VEGF mRNA to determine whether VEGF expression could directly influence the osteogenic potential of the transfected cells themselves. Further studies should include parallel experiments comparing direct mRNA transfection, conditioned medium, and rhVEGF across all functional assays to further distinguish direct and paracrine effects and to evaluate the biological efficacy of the mRNA platform relative to recombinant protein therapy.

VEGF has been identified as an essential factor linking osteogenesis and angiogenesis [52, 53]. Angiogenesis plays a crucial role in successful tissue regeneration, and insufficient vascularization may compromise regenerative outcomes. Therefore, VEGF‐induced angiogenesis is considered an essential component of periodontal regeneration. In the present study, quantitative analysis demonstrated significantly greater mineral deposition in the VEGF mRNA group. However, only a limited number of osteogenic markers were evaluated. Future studies should include more comprehensive quantitative mineralization assays and evaluate additional osteogenic marker genes, including *osterix* (*OSX*), *collagen type I alpha 1* (*COL1A1*), and *osteopontin* (*OPN*), to further validate these findings.

Importantly, there is currently insufficient evidence to conclude that VEGF directly induces osteogenic differentiation. Previous in vivo studies suggest that the osteogenic effects of VEGF are primarily mediated indirectly through enhanced angiogenesis in the bone microenvironment [22, 52, 53]. In the present in vitro study, where angiogenesis does not occur, the observed increase in mineralization may be attributed to VEGF‐induced cell proliferation rather than a direct effect on osteogenic differentiation.

The CAM assay was performed to evaluate the angiogenic potential of VEGF translated from mRNA‐transfected PDLCs. The VEGF mRNA group exhibited a significant increase in blood vessel formation, characterized by a radial spoke‐wheel‐like vascular pattern surrounding the graft. Due to its thin, transparent, and highly vascularized membrane, the CAM model enables real‐time visualization and quantification of neovascularization through a window under a stereomicroscope. Consequently, it serves as a valuable intermediate model bridging in vitro cell culture studies and more complex in vivo animal models. Our findings showed that the VEGF protein secreted by mRNA‐transfected PDLCs significantly promoted blood vessel formation in the CAM. These results are consistent with a previous study showing that VEGF RNA‐transfected in MG‐63 osteoblast‐like cells seeded on a polycaprolactone scaffold also enhanced angiogenesis [41]. Although the CAM assay is a well‐established model for evaluating angiogenesis, it does not fully recapitulate the complex biological conditions of the periodontal tissues. Therefore, the angiogenic effects observed in the CAM model should be interpreted as supportive evidence rather than definitive proof of periodontal tissue regeneration.

Even though we found that modified VEGF mRNA transfection enhanced cell proliferation and angiogenesis, the absence of VEGF neutralizing antibody experiment means that the biological effects observed in this study cannot be conclusively attributed to VEGF alone. Other soluble factors released by transfected cells or indirect effects associated with the transfection process may also have contributed to the observed biological responses. Therefore, future studies incorporating VEGF‐blocking strategies are required to confirm these findings. It should also be noted that VEGF expression was evaluated only up to 72 h. Nevertheless, this duration is longer than that reported for direct administration of rhVEGF protein, which has a biological half‐life of ~30 min [49]. This finding suggests that mRNA may provide a greater therapeutic benefit than direct protein administration. Additionally, this transient expression may be advantageous for therapeutic applications as prolonged or uncontrolled VEGF overexpression could lead to undesirable biological effects.

The present study demonstrated that the transfection with modified VEGF mRNA induced VEGF production, which subsequently enhanced cell proliferation and may contribute to osteogenic differentiation and angiogenesis; however, these findings alone do not establish the feasibility of periodontal regeneration in vivo. Further studies evaluating the regenerative potential of VEGF mRNA in periodontal defect models, such as ligature‐induced periodontitis in animals, are warranted. In addition, efficient and clinically applicable delivery strategies or scaffolds must be optimized to protect mRNA from degradation, enhance local retention within periodontal defects, achieve controlled release with appropriate dosing, improve in vivo transfection efficiency, and minimize diffusion to surrounding tissues. Potential delivery systems, including lipid nanoparticles, collagen‐based scaffolds, hydrogel carriers, and membrane‐associated delivery platforms, could be considered for future in vivo studies.

In our preliminary experiments, rhVEGF at a concentration comparable to that measured in the conditioned medium elicited only minimal angiogenic responses in the CAM model. Therefore, a higher dose of rhVEGF (500 ng per disc) was selected as a positive control to provide a well‐established angiogenic response. Direct comparisons between conditioned medium containing mRNA‐derived VEGF and rhVEGF at equivalent concentrations as well as at physiologically relevant levels would be crucial for future in vivo investigations. In addition, the immune response associated with the modified VEGF‐A mRNA should be evaluated in animal models prior to clinical translation. Further studies are also necessary to identify optimal biological scaffolds, such as using lipid nanoparticles for delivering and sustaining mRNA at the treatment site. Moreover, the potential of combining modified VEGF‐A mRNA with other regenerative growth factors, such as BMP‐2, PDGF‐BB, and fibroblast growth factor‐2 (FGF‐2), also merits investigation. Ultimately, further studies on the integration of the mRNA platform with cell‐based therapy and biomaterial scaffolds may substantially enhance angiogenic outcomes and tissue regeneration, thereby facilitating the clinical translation of mRNA‐based therapies for periodontal regeneration.

## 5. Conclusion

Modified pseudouridine mRNA encoding vascular endothelial growth factor‐A (VEGF‐A) was successfully transfected into hPDLCs, resulting in sustained VEGF production without significantly affecting cell viability or inducing proinflammatory cytokines. The translated VEGF enhanced hPDLC proliferation and osteogenic activity. Furthermore, it induced microvessel formation in the CAM assay. Taken together, these findings demonstrate the feasibility of applying a pseudouridine‐modified VEGF‐A mRNA platform in hPDLCs and provide a foundation for future in vivo studies investigating its potential for periodontal tissue regeneration.

## Author Contributions

**Noppadol Sa-Ard-Iam**: methodology (equal), investigation (lead), formal analysis (equal), writing – original draft (supporting), writing – review and editing (equal). **Mana Naratippakorn**: investigation (supporting), formal analysis (equal), writing – original draft (supporting). **Chantrakorn Champaiboon**: conceptualization (lead), funding acquisition (lead), methodology (equal), formal analysis (equal), writing – original draft (lead), writing – review and editing (equal).

## Funding

The study was supported by research funding from the Asian Research Center, Institute of Asian Studies, Chulalongkorn University, Thailand Science Research and Innovation Fund Chulalongkorn University (Grant CU_FRB65_hea (3) 009_32_04), and the Program Management Unit for Human Resources & Institutional Development, Research and Innovation‐CU (Grant B05F630075).

## Disclosure

All authors have read and approved the final version of the manuscript. Chantrakorn Champaiboon had full access to all of the data in this study and takes complete responsibility for the integrity of the data and the accuracy of the data analysis.

## Ethics Statement

The human study was conducted in accordance with the Declaration of Helsinki (1964). The in vitro study protocols were approved by the Human Research Ethics Committee (HREC‐DCU 2020‐063, 2021‐071, and 2022‐018) and the Institutional Biosafety Committee (DENT CU‐IBC Numbers 021/2020 and 0621.06/2935) of the Faculty of Dentistry, Chulalongkorn University, Thailand. Written informed consent was obtained from all participants prior to study procedures. The animal care and use protocol was approved by the Chulalongkorn University Animal Care and Use Committee (VET‐ACUP Number 2031021‐Renew) of the Faculty of Veterinary Science, Chulalongkorn University, Thailand.

## Conflicts of Interest

The authors declare no conflicts of interest.

## Data Availability

The data supporting the findings of this study are available from the corresponding author upon reasonable request.
